# Impacts of Divorce and Widowhood on Depressive Symptoms Before and During the COVID-19 Pandemic

**DOI:** 10.1093/geroni/igaf122.1132

**Published:** 2025-12-31

**Authors:** Erin Bisesti, Haowei Wang

**Affiliations:** Syracuse University, Syracuse, New York, United States; Syracuse University, Syracuse, New York, United States

## Abstract

The landscape of marital dissolution among older adults has been evolving over the last several decades in the U.S., with high and rising divorce rates and increased risk of losing a spouse during the COVID-19 pandemic. Older adults experienced more loneliness following the marital dissolution, leading to potentially greater risk of depression. We used data from the 2016-2020 Health and Retirement Study to examine the relationship between marital dissolution and depression before and during the COVID-19 pandemic. Marital status included remained married, remained divorced, remained widowed, became divorced, and became widowed between two consecutive waves (2016-2018 and 2018-2020). Depression was measured using a cutpoint of 3 or more out of eight CES-D symptoms. Our final sample included 12,432 participants aged 50+. In 2018, 15.35% of participants reported depression compared to 20.90% in 2020. We performed logistic regressions estimating the effect of marital dissolution on depression. We found that, compared to those who remained married, all other categories had higher odds of reporting depression. Specifically, the odds of reporting depression were the highest among those who became widowed (OR = 5.24) or divorced (OR = 4.62) between interview waves. This pattern was consistently found in the pre-pandemic (2016-2018) and during the pandemic (2018-2020). Additional analysis found that remaining divorced during 2020 was less detrimental to depression than remaining married (OR = 0.72). Findings suggest that mental health services should be more readily available to older adults after the loss of a spouse through divorce or widowhood.

